# Planting Healthier Indoor Air

**DOI:** 10.1289/ehp.119-a426

**Published:** 2011-10-01

**Authors:** Luz Claudio

**Affiliations:** Luz Claudio, PhD, is a tenured associate professor in the Department of Preventive Medicine at Mount Sinai School of Medicine in New York City. Her research focuses on global environmental health.

Poor indoor air quality has been linked to health problems, especially in children. Asthma has reached epidemic proportions among multiple age groups and is considered the most common chronic disease in urban-dwelling children.^1^ The American Academy of Allergy, Asthma and Immunology Indoor Allergen Committee suggested in a 2010 report that allergists consider indoor air filtration to be part of a comprehensive strategy to improve respiratory health.^2^ Air cleaners with HEPA filters have been shown to improve symptoms of asthma.^2^ However, filtration systems and air purifiers do not reduce levels of all indoor air pollutants, and some types can actually aggravate the problem. For example, one study showed that some air purifiers raise indoor concentrations of ozone above safety levels established by the U.S. Environmental Protection Agency.^3^

A more benign addition to air filtration could be the use of houseplants. In addition to basic photosynthesis that removes carbon dioxide and returns oxygen to the air, plants can remove toxicants from air, soil, and water in at least two ways. First, they can metabolize some toxic chemicals, releasing harmless by-products, and second, they can incorporate toxicants such as heavy metals into plant tissues, thus sequestering them.

Data on plant-mediated indoor air quality come from experiments conducted by the U.S. National Aeronautics and Space Administration (NASA). As NASA researchers explored the possibilities of long-term space habitation, it became evident that the air in a tightly sealed space capsule would quickly become contaminated with volatile organic compounds (VOCs) and other chemicals released by the materials used to manufacture the capsule interior.^4^

This is similar to the situation in newly constructed energy-efficient dwellings. If energy-efficient construction is not carefully designed to maintain indoor–outdoor air exchange, one unintended consequence can be increased concentrations of pollutants indoors. For example, in a study recently published in the *American Journal of Public Health*, Gary Adamkiewicz and colleagues used a simulation model to demonstrate that in homes with low air exchange rates and multiple sources of air pollution, up to 90% of exposure to fine particulate matter came from indoor sources.^5^ Besides particles and VOCs, indoor air and dust can also contain brominated flame retardants, pesticides, toxic metals, and other pollutants.^6^

For more than 30 years, B.C. “Bill” Wolverton, a retired civilian scientist for NASA, investigated the use of plants as air- and water-purifying systems for enclosed environments in space missions. Through his research, Wolverton found the air-cleaning capacity of houseplants can be improved exponentially by increasing air circulation to the roots of the plants, where symbiotic microorganisms help make the substances culled from air bioavailable to the plant.

**Figure:**
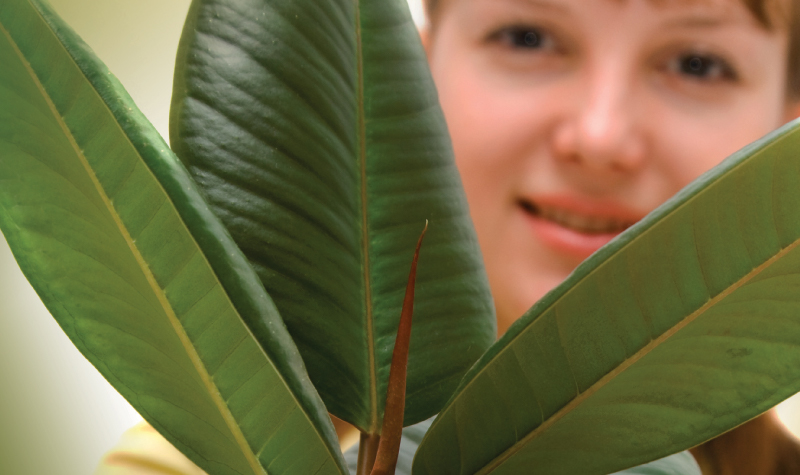
For maximum benefit, multiple species of houseplants would likely be needed on a site to remove the relevant toxicants in a particular space, given that houseplants vary in the types of chemicals they are able to remove from the environment and the efficiency with which they do their work. “For effective phytoremediation, the number and type of plants selected would need to be tailored to each individual building,” says Stanley J. Kays of the University of Georgia. Shutterstock.com

In those studies, Wolverton and colleagues tested several types of low-light houseplants.^7^ For example, golden pothos (*Epipremnum aureum*, also known as devil’s ivy) grown on an activated carbon filter system reduced air levels of benzene and trichloroethylene inside a Plexiglas chamber measuring 0.58 cubic yard from approximately 36 ppm to barely detectable levels within 2 hours.^4^ Experiments conducted elsewhere by Stanley J. Kays and colleagues at the University of Georgia also documented the ability of different plant species to remove VOCs such as benzene, toluene, octane, and trichloroethylene.^8^

One indoor contaminant of particular concern is formaldehyde, which is released by many household products, among them pressed woods, some types of foam insulation, paper products, some paints and varnishes, and permanent-press fabrics. The National Toxicology Program lists formaldehyde as reasonably anticipated to be a human carcinogen.^9^

In an unpublished 2006 study, Wolverton tested a small fan-assisted planter/air filter inside a travel trailer that had been used as temporary housing for displaced Hurricane Katrina victims. This trailer, like similar units, had been found to be highly contaminated with formaldehyde. The plant/air filter contained a plant growing in a mixture of activated carbon and expanded clay pebbles. Wolverton’s tests showed that the levels of formaldehyde were reduced from potentially toxic levels of 0.18 ppm to 0.03 ppm, within the safety limits defined by the World Health Organization.^10^

Those studies fit well with evidence on the biochemical mechanisms involved in plant detoxification of formaldehyde. In studies published this year Zhongjun Xu and colleagues tested three kinds of potted plants for their capacity to remove formaldehyde from indoor air in test chambers. They found that the formaldehyde-removal capacity of the plants depended on the dehydrogenase activity in the leaves and root system—that is, how efficiently the plant could metabolize formaldehyde.^11^ As Wolverton found earlier, these investigators also found that formaldehyde removal by plants was diffusion-limited. That means increasing the circulation of contaminated air through the root system and leaves improved the formaldehyde-removal effect.

**Figure:**
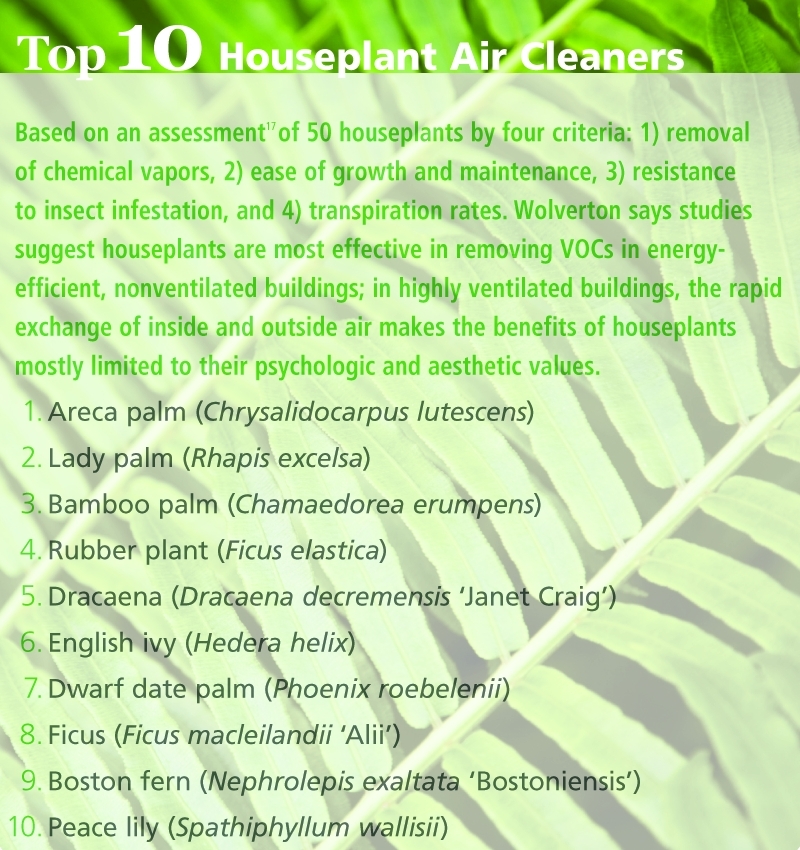
**Top 10 Houseplant Air Cleaners** Based on an assessment^17^ of 50 houseplants by four criteria: 1) removal of chemical vapors, 2) ease of growth and maintenance, 3) resistance to insect infestation, and 4) transpiration rates. Wolverton says studies suggest houseplants are most effective in removing VOCs in energyefficient, nonventilated buildings; in highly ventilated buildings, the rapid exchange of inside and outside air makes the benefits of houseplants mostly limited to their psychologic and aesthetic values. Shutterstock.com

In another recent study, Kays and colleagues tested 86 species of houseplants from five general classes for their ability to remove formaldehyde. In their experiments, ferns had the highest formaldehyde-removal efficiency of all the plants tested, especially *Osmunda japonica*, commonly known as Japanese royal fern, or zenmai.^12^

Another important air contaminant that is amenable to plants’ cleanup abilities is mercury vapor. Mercury can make its way into homes through accidental spills (for instance, breakage of thermometers and fluorescent bulbs) as well as through its use in certain cultural and religious practices.^13^ Mercury vapor is neurotoxic and lingers in the air even after new sources have been eliminated from the environment.^14^

Joao Paulo Machado Torres, a senior scientist at the Radioisotopes Laboratory of the Federal University of Rio de Janeiro, Brazil, and his group have published many studies on the use of plants in indoor and outdoor mercury-contaminated settings.^15^ “We have used plants of the bromeliad family and Spanish moss (*Tillandsia usneoides*) as sentinel species to detect and absorb mercury from the air in shops contaminated by the gold trade in the Amazon,” he says. The use of plants can be uniquely useful in these environments where other kinds of remediation technology may be impractical or difficult to deploy.

But as has been shown with many natural remedies, “natural” does not necessarily equate to “absolutely harmless.” A study by Kays and colleagues published in 2009 pointed out that some houseplants—as well as the media and plastic pots they are grown in, the microorganisms that inhabit them, and the pesticides used to treat them—can potentially contaminate indoor air with VOCs.^16^ “It is not yet possible to project the true potential of plants for purifying indoor air,” Kays says. “At this time the role of plants, though appearing [generally] positive, is not totally clear. The absence of funding for phytoremediation research has greatly impeded solving the problem.”

Kays also notes the lack of an accurate means for the public to determine if the VOCs in their home or office represent a significant health problem. “The absence of a relatively inexpensive method available to the public results in situations where it takes two and a half years to determine that the Katrina trailers had toxic levels of formaldehyde even though there had been health complaints by the occupants almost as soon as the trailers were in place,” he says. “If an accurate, reasonably priced method was available from a credible source such as a university extension analytical laboratory, the public would be able to ascertain their potential health risk before buying or renting a house, apartment, or office.”
